# Medium to long term follow-up of survival and quality of life in patients with primary tumors of the cervical spine: Experience From a large single center

**DOI:** 10.3389/fsurg.2022.1011100

**Published:** 2023-01-06

**Authors:** Nanfang Xu, Shuai Chang, Xiaoguang Liu, Liang Jiang, Miao Yu, Fengliang Wu, Lei Dang, Hua Zhou, Yan Li, Yongqiang Wang, Xiao Liu, Yunxia Wu, Feng Wei, Zhongjun Liu

**Affiliations:** ^1^ Department of Orthopedics, Peking University Third Hospital, Beijing, China; ^2^ Beijing Key Laboratory of Spinal Disease Research, Peking University Third Hospital, Beijing, China; ^3^ Engineering Research Center of Bone and Joint Precision Medicine, Ministry of Education, Peking University Third Hospital, Beijing, China

**Keywords:** primary tumors of cervical spine, primary cervical spinal tumors, surgical treatments, health-related quality of life (HRQoL), EQ-5D, survival, risk factors

## Abstract

**Objectives:**

To evaluate the survival and medium to long term health-related quality of life (HRQoL) of patients with primary cervical spinal tumors in a cross-sectional study and to identify any significant associations with demographic or clinical characteristics.

**Methods:**

Patients diagnosed with primary cervical spinal tumors were retrospectively enrolled and their clinical, radiologic, and follow-up data (specifically the EQ-5D questionnaire) were collected. Univariate and multivariate Cox time-dependent regression analyses were performed to examine the significance of certain variables on overall survival. Univariate and multivariate logistic regression analyses were conducted to identify variables significant for overall HRQoL and each dimension of the EQ-5D.

**Results:**

A total of 341 patients were enrolled in the study with a mean follow-up of 70 months. The diagnosis was benign in 246 cases, malignant in 84, and unconfirmed in 11. The 5-year overall survival rate was 86% and the 10-year overall survival rate was 65%. Multivariate analysis suggested that surgical treatment (*P* = 0.002, hazard ratio [HR] = 0.431, 95% CI. [0.254, 0.729]), benign and malignant tumors [*P* < 0.001, HR = 2.788, 95% CI. (1.721, 4.516)], tumor and surrounding normal tissue boundary [*P* = 0.010, HR = 1.950, 95% CI. (1.171, 3.249)], and spinal instability [*P* = 0.031, HR = 1.731, 95% CI. (1.051, 2.851)] still had significant effects on survival.

**Conclusions:**

In this cross-sectional study, we evaluated the survival period and medium and long-term health-related quality of life of patients with primary tumors of the cervical spine, and analyzed the significant related factors of tumor clinical characteristics. Surgery, myelopathy, malignancy, spinal pain relieved by lying down or supine position, and tumor infiltration on MRI were significant predictors for overall survival. Enneking stage and age were significant predictors for HRQoL.

## Introduction

Primary tumors of the cervical spine are relatively rare, with a global incidence rate of approximately 2.5–8.5 cases per 100,000 people per year (1). Primary cervical spinal tumors account for no more than 10% of spinal tumors (2) and 2.8%–13% of primary bone tumors (3). In comparison, the annualized incidence rates for the common spinal surgery diseases of lumbar spinal stenosis, cervical spondylotic radiculopathy, and acute spinal cord injury are 3000, 830, and 50 cases per 100,000 people, respectively (1). Primary spinal tumors were generally benign in children (60%) and malignant in adults (80%) (4).

Although the incidence of primary cervical spinal tumors is low, the mortality and disability rates are high (5), especially among young patients (6). The main causes of death or disability in affected patients are local tumor invasion of adjacent structures, nerve damage caused by nerve compression, and systemic metastasis (7). Owing to the extremely low incidence rate, current treatments are mostly based on case series studies and summaries, and lack high-quality evidence (5, 8–15).

The purpose of the present study was to clarify the treatment and prognosis of patients with primary cervical spinal tumors in the Department of Orthopedics, Peking University Third Hospital during a 20-year period (1994–2014). By collecting survival data for all patients with and without surgical treatment, and obtaining HRQoL data for the surviving patients by medium and long-term cross-sectional follow-up (through the EQ-5D questionnaire), we intended to identify factors with significant impacts on the survival and HRQoL of the patients, and ultimately to provide references for the diagnosis, treatment, and prognosis of primary cervical spinal tumors in the future.

## Methods

### Study participants: Screening for primary cervical spinal tumor cases

**Figure 1:**
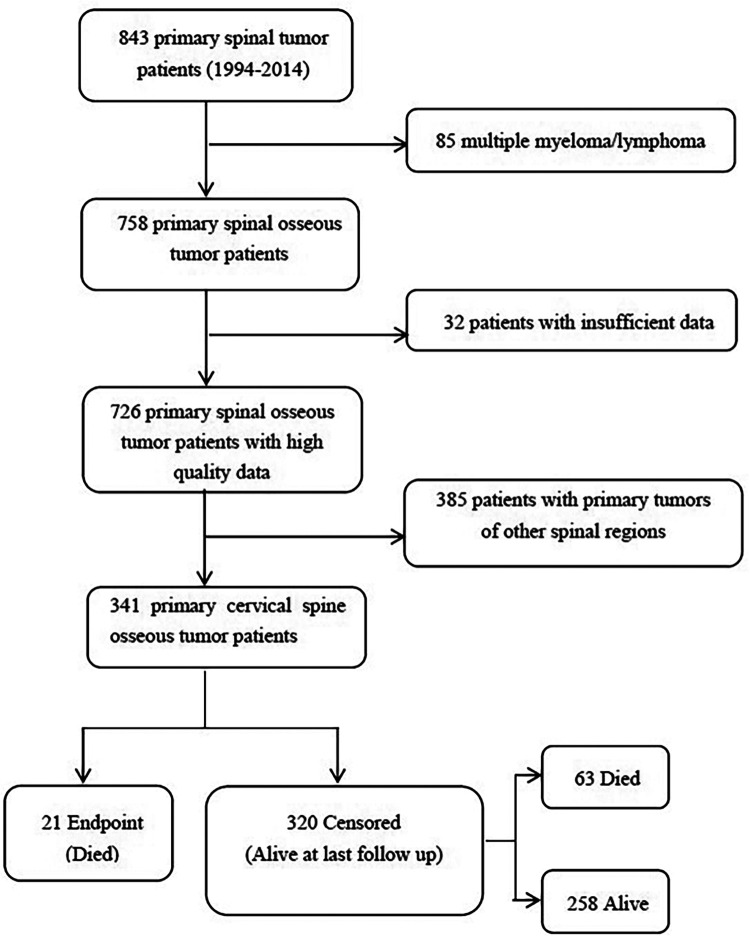
Screening process for the 341 patients with primary cervical spinal tumors.

The study was approved by the Peking University Third Hospital Ethics Committee (IRB approval number: 12–13-QX-GIC). The study retrospectively collected and compiled data for all patients with primary cervical spinal tumors from 1994 to 2014 in the Department of Orthopedics, Peking University Third Hospital, and individually checked the patients in the hospital's medical record system database to confirm their final diagnosis and parts affected by the disease. The study excluded duplicate cases and cases with non-neoplastic diseases. In addition, the cases of primary tumors in other spinal regions, lymphoma, multiple myeloma, and plasmacytoma not resulting from spinal invasion, were also removed by us. Ultimately, we selected the cases with lesions located in the upper cervical spine (C1–C2) and subaxial cervical spine (C3–C7) (Figure 1).

### Clinical data: Clinical data extraction for primary cervical spinal tumor cases

The study retrieved the contents of the hospital medical records for 341 primary cervical spinal tumor cases and extracted their clinically relevant data. The above data included five aspects: general clinical characteristics indexes, symptomatic indexes, spinal cord function score indexes, tumor clinical stage indexes and related indexes of the surgeries. General clinical characteristics indexes included age, sex, date of birth, ID number, address, telephone number, date of admission, date of discharge, date of operation, final diagnosis, lesion location (upper or subaxial cervical spine) and surgical history for lesion in other hospitals. Symptomatic indexes included time of symptom onset, spinal pain, nocturnal pain, whether pain could be relieved by lying down or supine position, radiating pain, whether there were symptoms of spinal cord injury (SCI) such as arm and leg muscle weakness and/or paresthesia, whether there were local masses in the lesion (such as palpable neck masses, foreign body sensation when swallowing) and whether neural control of bladder, bowel, and sexual function were affected. Spinal cord function score indexes included preoperative ASIA score (grades A through E for spinal cord injury indicate gradual alleviation, where E reflects no motor and sensory impairment). Tumor clinical stage indexes included Enneking stages (benign tumors were classified as S1, S2, S3; malignant tumors were classified as I, II, III, and further classified as IA, IB, IIA, IIB, III depending on whether the lesions were confined in a compartment). Related indexes of the surgeries included surgical treatments, surgical approaches (anterior, posterior, anterior followed by posterior, posterior followed by anterior, posterior followed by simultaneous anterior, anterior followed by simultaneous posterior, staged posterior-anterior combined), surgical procedures (en-bloc excision, curettage, and palliative or debulking surgery, including decompression and fixation) and surgical boundaries (intralesional excision, surgical resection with negative margins, and extensive excision).

### Patient follow-up

The study collected follow-up data for all patients with primary tumors of the cervical spine. Based on the survival status at the last follow-up, the cases were assigned to the endpoint group (dead) or the non-endpoint group (surviving). For cases in the endpoint group, the date of death and cause of death, such as tumor recurrence, tumor metastasis, complications caused by tumor treatment, or non-tumor-related reasons, were recorded. For the cases in the non-endpoint group, a standardized follow-up script was used to conduct cross-sectional follow-up according to the contact information for the patients and their families in the medical record system from June to July 2015. The patients were asked whether they had any complaints of discomfort at present, and their current HRQoL was assessed by the EQ-5D questionnaire. Due to the age range for the EQ-5D Questionnaire is 8–80 year, pediatric forms (under 16) of the questionnaire also were provided in the study.

### Statistical analysis

Descriptive statistical analyses were conducted for the clinical and follow-up data, and the overall incidence rate of each tumor type and the incidence rates in different age groups were calculated and compared. The 5- and 10-year overall survival rates and the mean HRQoL scores were calculated. The continuous variables were discretized as follows: age was divided into two grades, ≤55 years and >55 years; Enneking stage was divided into three grades, S1, S2–S3, and I–III; and the ASIA score classification of spinal cord injury (SCI) can be divided into five levels, A, B, C, D and E.

A Cox time-dependent model was used to analyze the significant influencing factors for survival, and a logistic regression model was used to analyze the significant influencing factors for HRQoL. The relevant statistical analyses were performed using SPSS 21.0 software (IBM Corp., Armonk, NY, USA).

## Results

### Case screening results

The study identified a total of 843 patients with primary cervical spinal tumors at the Department of Orthopedics, Peking University Third Hospital, between 1994 and 2014. After excluding 117 cases with multiple myeloma, lymphoma, and incomplete case data, 726 patients were enrolled in the study, including 341 cases with primary cervical spinal tumors, accounting for 47% of the total primary spinal tumor cases in the hospital (summarized in Figure 1).

### Follow-up data

Of the 341 patients with primary tumors of the cervical spine, 21 were assigned to the endpoint group (died during follow-up, including 18 with surgery and 3 without surgery), and 320 were assigned in the non-endpoint group (survival at last follow-up). In the non-endpoint group, 225 patients (70.3%) or their families were successfully followed up by telephone from June to July 2015. Of these, 187 (83.1%) survived (137 with surgery and 50 without surgery) and 38 (16.9%) died (27 with surgery and 11 without surgery). Meanwhile, 95 patients (29.7%) had survival information retrieved from the internal computer system of the Public Security Bureau in August 2015, of whom 71 (74.7%) survived (55 with surgery and 16 without surgery) and 24 (25.3%) died (16 with surgery and 8 without surgery).

### Summary of clinical and follow-up data

Of the 341 cases, 330 (97%) were diagnosed by biopsy or surgical pathology. Of the remaining cases, 3 (1%) had surgical pathology that could not determine the specific tumor type, including 2 benign cases and 1 malignant case, and 8 (2%) did not undergo biopsy or surgery and thus had unknown specific diagnosis, although their clinical evaluations were benign. Among all 341 cases, 256 (75%) had benign tumors and 85 (25%) had malignant tumors, 211 cases had subaxial cervical spinal (C3–C7) tumors (62%) and 130 cases had upper cervical spinal (C1–C2) tumors (38%), there were 201 males (59%) and 140 females (41%), and the mean age was 35 years.

The number of cases for each tumor type, ratio of each tumor type among all primary cervical spinal tumors, sex ratio for each tumor type, and location ratio for each type of tumor (upper or subaxial cervical spine) are shown in Table 1.

**Table 1:** Numbers and proportions of cases corresponding to different tumor types.

Diagnosis	# of patients	Percentage	Gender (M/F)	Location (AA/SA)	Average age (yr)
Benign	246	72.1	149/97	83/163	31.4
Schwannoma	61	17.9	38/23	16/45	44.4
EG	44	12.9	29/15	20/24	14.2
GCT	35	10.3	15/20	11/24	31.9
Osteoblastoma	19	5.6	15/4	4/15	22.8
ABC	15	4.4	9/6	6/9	26.8
Osteoid osteoma	14	4.1	8/6	6/8	23.8
Osteochondroma	12	3.5	5/7	3/9	25.3
FD	9	2.6	8/1	6/3	34.3
Hemangioma	8	2.3	4/4	2/6	46.9
Neurofibroma	7	2.1	6/1	2/5	40.4
Tenosynovial GCT	6	1.8	2/4	3/3	31.8
Fibromatosis	4	1.2	3/1	0/4	46.8
Ganglioma	4	1.2	2/2	1/3	52.5
Hemangiopericytoma	3	0.9	3/0	2/1	38.3
Hemangioendothelioma	2	0.6	1/1	0/2	24.0
Lipoma	1	0.3	0/1	0/1	45.0
Hemangioblastoma	1	0.3	1/0	0/1	55.0
Myofibroblastoma	1	0.3	0/1	1/0	4.0
Malignant	84	24.6	48/36	37/47	46.1
Chordoma	38	11.1	21/18	20/19	45.8
Plasmacytoma	18	5.3	10/8	9/9	53.0
Chondrosarcoma	8	2.3	4/4	2/6	42.0
PNET	7	2.1	6/1	2/5	25.7
MPNST	5	1.5	3/2	2/3	54.6
Osteosarcoma	4	1.2	1/3	0/4	50.8
Malignant solitary fibrous tumor	2	0.6	2/0	1/1	47.0
Synovial sarcoma	1	0.3	1/0	1/0	29.0
Unconfirmed	11	3.2	4/7	10/1	36.0
Total	341	100	201/140	130/211	35.1

Professional term abbreviations: ① EG, eosinophilic granuloma; ② GCT, giant cell tumor of bone; ③ ABC, aneurysmal bone cyst; ④ FD, fibrous dysplasia; ⑤ PNET, peripheral neuroectodermal tumor; ⑥ MPNST, malignant peripheral nerve sheath tumor.

The total patients were divided into seven age groups: 0–10, 11–20, 21–30, 31–40, 41–50, 51–60, and >60 years. The number of patients in each group, proportion of patients in each group relative to the total patients, sex ratio of patients in each group, proportions of benign and malignant tumors in each group, and proportions of tumor location (upper or subaxial cervical spine) in each group are shown in Table 2.

**Table 2:** Sex, benign and malignant tumors, and tumor locations in different age groups.

Decade	# of patients	Gender% (M/F)	Age%	Malignancy% (B/M)	Location% (AA/SA)
0–10	46	6.7/6.7	13.5	12.9/0.6	6.7/6.7
11–20	41	8.5/3.5	12.0	10.9/1.2	5.9/6.2
21–30	56	8.8/7.6	16.4	13.5/2.9	5.9/10.6
31–40	58	11.1/5.9	17.0	14.1/2.9	6.5/10.6
41–50	63	11.1/7.3	18.5	12.6/5.9	5.6/12.9
51–60	41	5.6/6.5	12.0	5.3/6.7	4.4/7.6
61 and up	36	7.0/3.5	10.6	5.6/5.0	3.2/7.3
Total	341	58.9/41.1	100	74.8/25.2	38.1/61.9

The mean time from symptom onset to treatment was 13 months. Thirty-five cases (10%) had recurrence after one-stage surgical resection of tumors in other hospitals, and 39 (11%) received preoperative adjuvant radiotherapy. Another 154 cases (45%) received chemotherapy after diagnosis, while 86 cases (25%) received neoadjuvant chemotherapy, the remaining 68 cases (20%) received postoperative chemotherapy. Neck and shoulder pain (spinal pain) was the most common symptom (274 cases, 80%), of which 99 cases (29%) could be relieved by lying down, 66 (19%) were radiating pain, and 57 (17%) were aggravated at night. Meanwhile, 132 cases (39%) manifested SCI such as arm and leg muscle weakness and/or paresthesia, of which 20 cases (6%) had bowel/bladder involvement, while 38 (11%) had palpable neck mass or dysphagia, and 184 cases (54%) had clear boundaries between the tumor and surrounding normal tissues on MRI. According to the ASIA classification of SCI, the proportions of patients with grades A, B, C, D, and E (gradual reduction in SCI) were 0.9%, 3.5%, 4.7%, 29.9%, and 61.0%, respectively. Regarding the Enneking stages, the proportions of patients with S1, S2, and S3 were 17.6%, 30.5%, and 23.5%, respectively, and the proportions of patients with IA, IB, IIA, IIb, and III were 3.2%, 13.2%, 2.3%, 8.2%, and 1.5% respectively. The relevant data for all patients, surgical patients, and non-surgical patients are summarized in Table 3.

**Table 3:** Summary of the clinical data for the patients.

	All patients	Surgical patients	Non-surgical patients
No. of patients	341	253	88
Mean age (yr)	35.1	38.5	25.3
Gender% (M/F)	58.9/41.1	58.1/41.9	61.4/38.6
Malignancy% (B/M)	74.8/25.2	72.3/27.7	83.0/17.0
Location% (AA/SA)	38.1/61.9	32.0/68.0	55.7/44.3
Prev surg%	10.0	12.6	3.4
Duration of symptoms (mo)	13.0	13.7	11.2
Spinal pain%	80.6	76.3	92.0
Night pain%	16.8	17.0	15.9
Motion-related pain%	29.1	22.9	46.6
Radicular pain%	19.4	20.9	14.8
Weakness/Paresthesia%	38.5	44.7	21.6
Bowel/Bladder involvement%	5.9	7.5	1.1
Tumor mass%	11.2	9.9	14.8
Tumor invasion%			
Confined	54.1	47.8	71.6
Invasive	45.9	52.2	71.6
Enneking%			
S1	17.6	6.4	68.6
S2	30.5	35	19.6
S3	23.5	28.1	11.6
IA	3.2	4	1.2
IB	13.2	15.2	8.1
IIA	2.3	3.2	0.0
IIB	8.2	9.6	4.6
III	1.5	0	5.8
ASIA%			
A	0.9	1.2	0.0
B	3.5	4.3	1.1
C	4.7	5.1	3.4
D	29.9	34.4	17.0
E	61.0	54.9	78.4

Our surgical indications are basically summarized in the following two points. Firstly, malignancy is once diagnosed. Secondly, symptoms of spinal cord compression occur, regardless of benign or malignant tumors. Among the total 341 cases, 253 (74%) were treated using surgical approaches, including 53 (16%) with anterior approach, 95 (28%) with posterior approach, 33 (10%) with posterior followed by anterior approach, 29 (9%) with anterior followed by posterior approach, 35 (10%) with posterior followed by simultaneous anterior approach, and 8 (2%) with staged posterior-anterior combined approaches. Another 88 cases (25.8%) did not undergo surgery and they underwented a needle biopsy and thus obtained a histological diagnosis.

Among the 253 cases with surgical boundaries, 109 (32%) were intralesional excision, 91 (27%) were surgical resection with negative margins, and 45 (13%) were extensive excision; In terms of surgical procedures, 181 cases (53%) underwent piecemeal total or subtotal excision or curettage, 64 (19%) underwent en-bloc excision; And 8 (2%) underwent palliative or debulking surgery (including decompression and fixation). Of the 253 surgical patients, the purpose of were treated with fusion was to increase stability, therefore only 45 (13%) patients who were extensive excision were treated with fusion.

### Analysis of survival and its influencing factors

**Figure 2:**
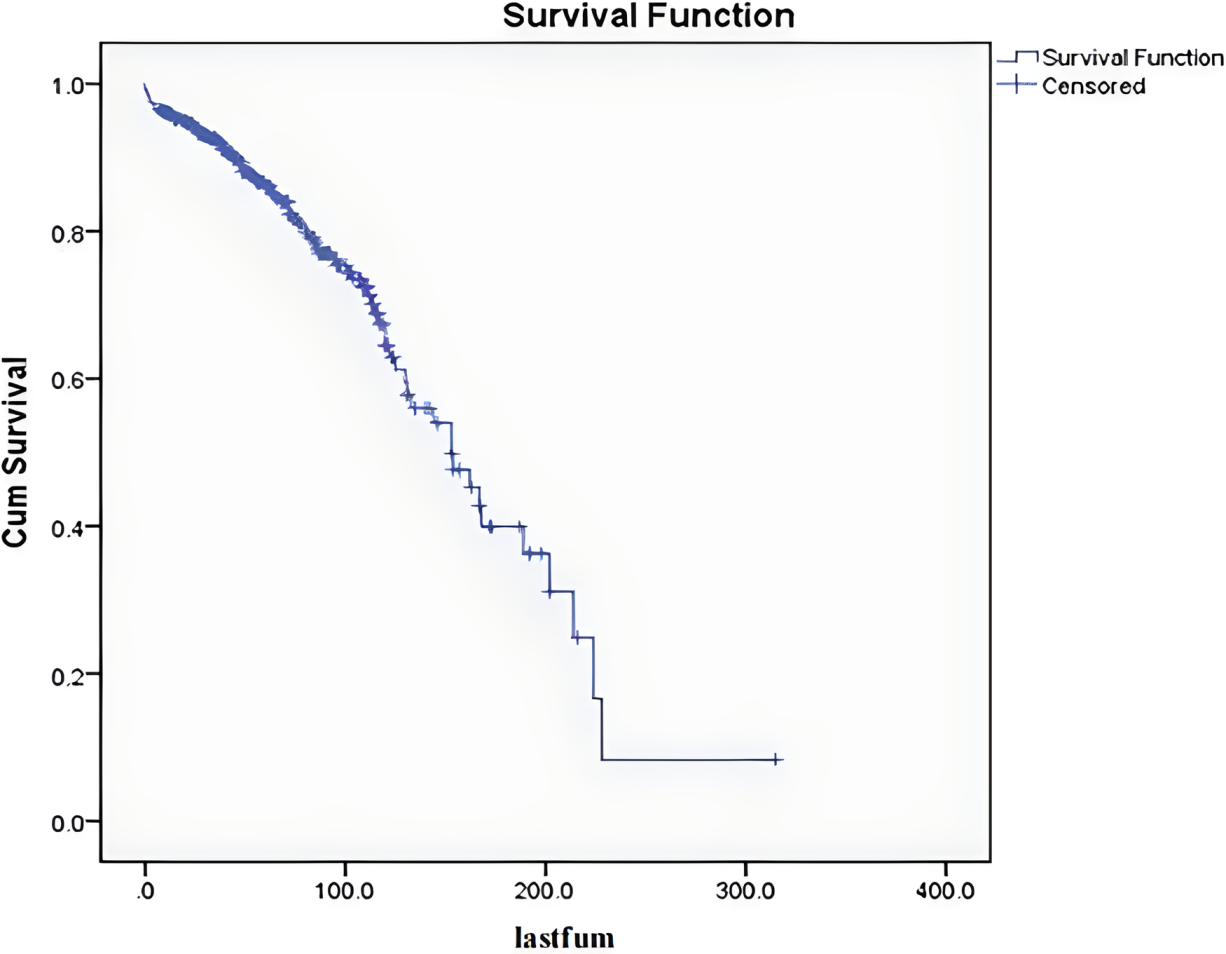
Overall survival curve for the 341 patients.

Among the 341 cases, the mean follow-up period was 70 months. After excluding the 12 patients who died within 5 months after surgery (The cause of death in the 12 patients who died within 5 months after surgery was unrelated to tumor/operation, three from pulmonary embolism, five from pulmonary infection with respiratory failure, and four from cardiovascular and cerebrovascular disease.), the follow-up period ranged from 7 to 198 months. The 5-year overall survival rate was 86%, the 10-year overall survival rate was 65%, and the median survival period was 153 months (Figure 2).

Univariate analyses with the Cox time-dependent model showed that the significant risk factors for survival were surgical treatment [*P* = 0.013, HR = 0.547, 95% CI. (0.340, 0.881)], benign and malignant tumors [*P* < 0.001, HR = 3.103, 95% CI. (1.998, 4.821)], tumor and surrounding normal tissue boundary [*P* = 0.004, HR = 1.941, 95% CI. (1.240, 3.037)], spinal instability [*P* = 0.004, HR = 1.942, 95% CI. (1.235, 3.055)], and tumor location (upper spine or subaxial cervical spine) [*P* = 0.009, HR = 1.814, 95% CI. (1.161, 2.836)].

**Figure 3:**
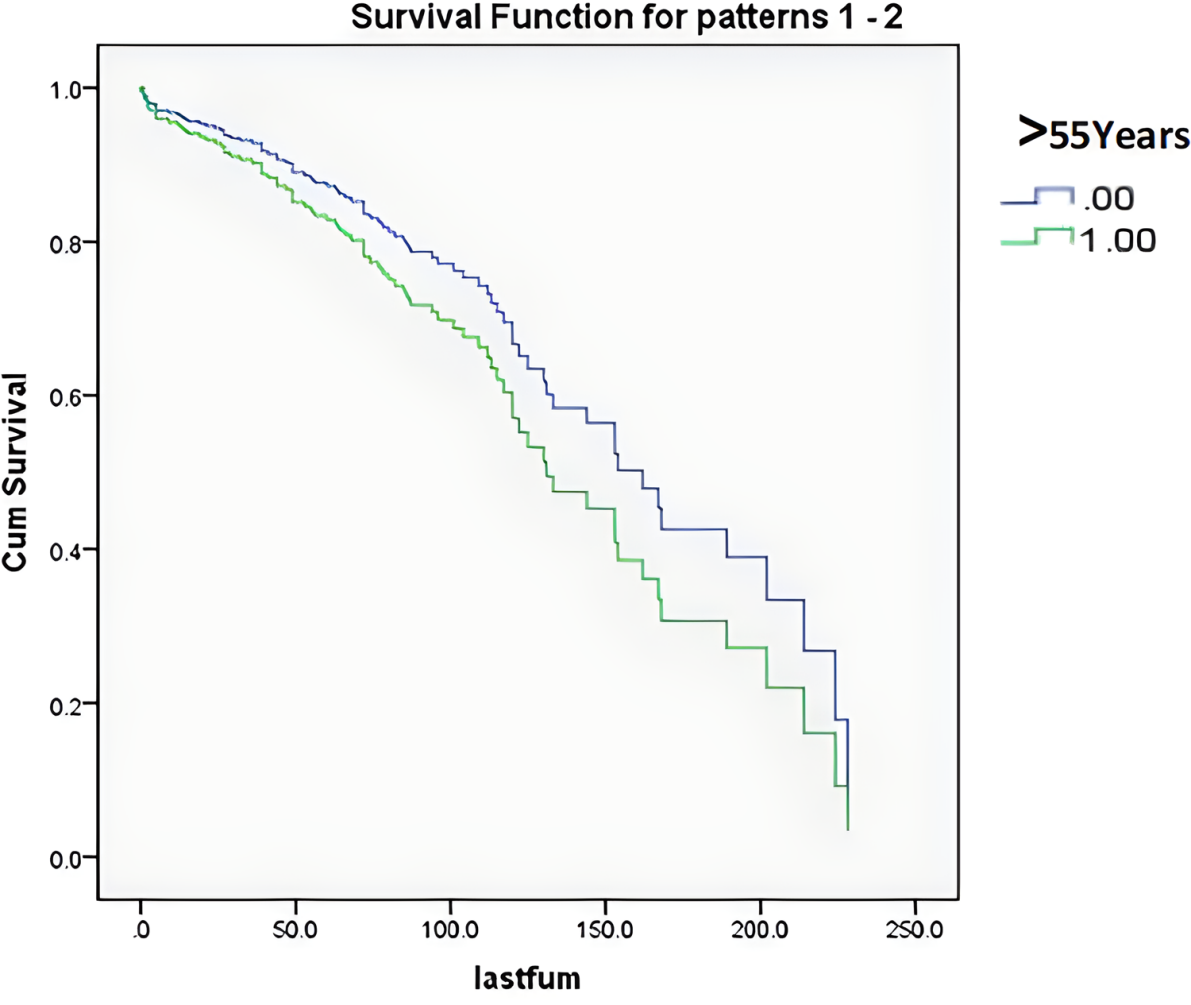
Survival curves of the patients discretized by age of 55 years.

**Figure 4:**
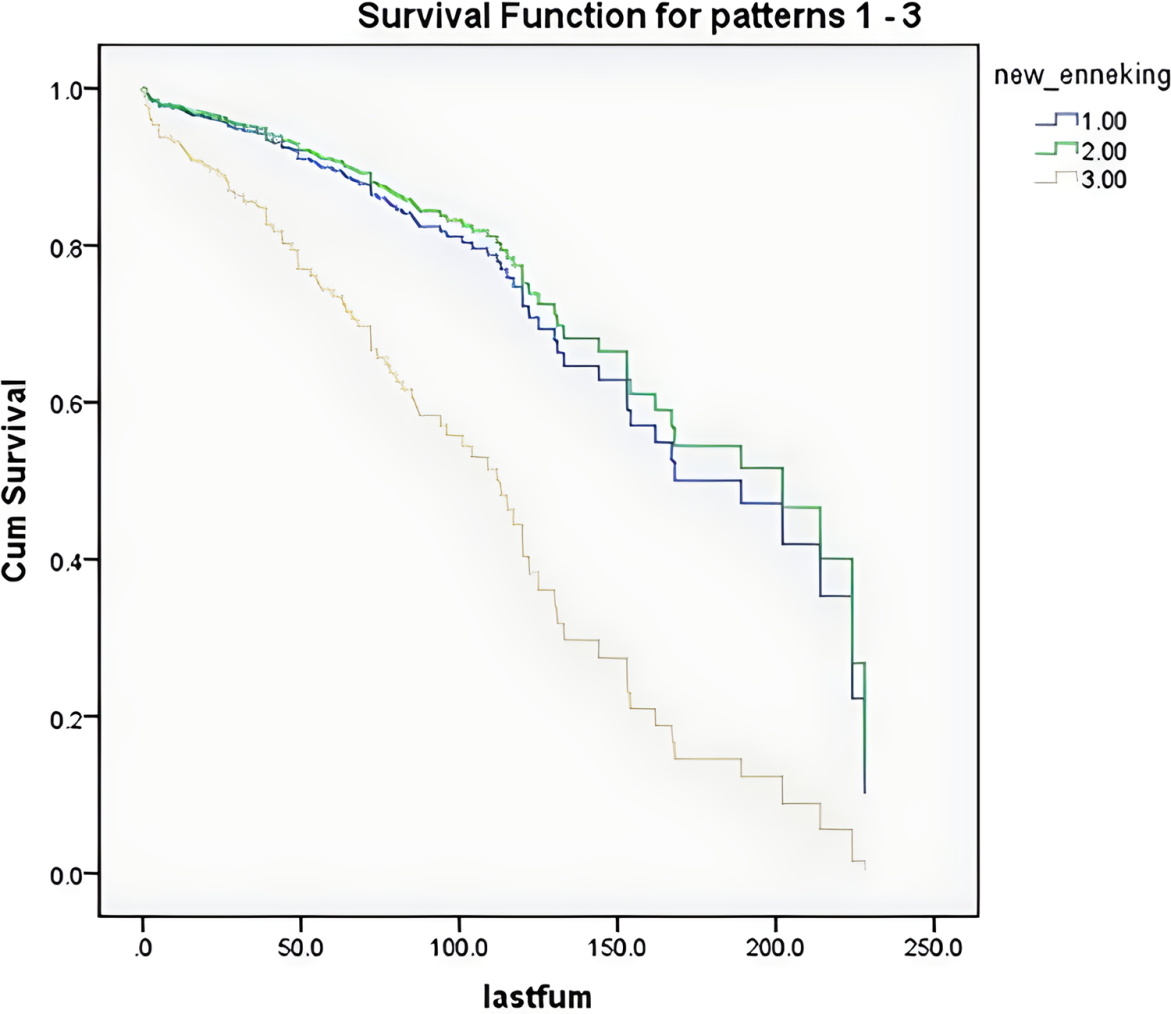
Survival curves of the patients after discretization by Enneking stages.

According to the survival curves for the age groups after discretization (Figure 3), the survival time of older patients was shorter, consistent with the clinical experience, but the results were not significant. According to the survival curves for the Enneking stages after discretization (Figure 4), there was no significant difference between stage S1 and stage S2–S3 patients, but there was a significant difference between these patients and stage I–III patients. These findings were consistent with the results that benign and malignant tumors were significant factors affecting survival time (both showed significant collinearity, correlation coefficient *r* = 0.94).

**Figure 5:**
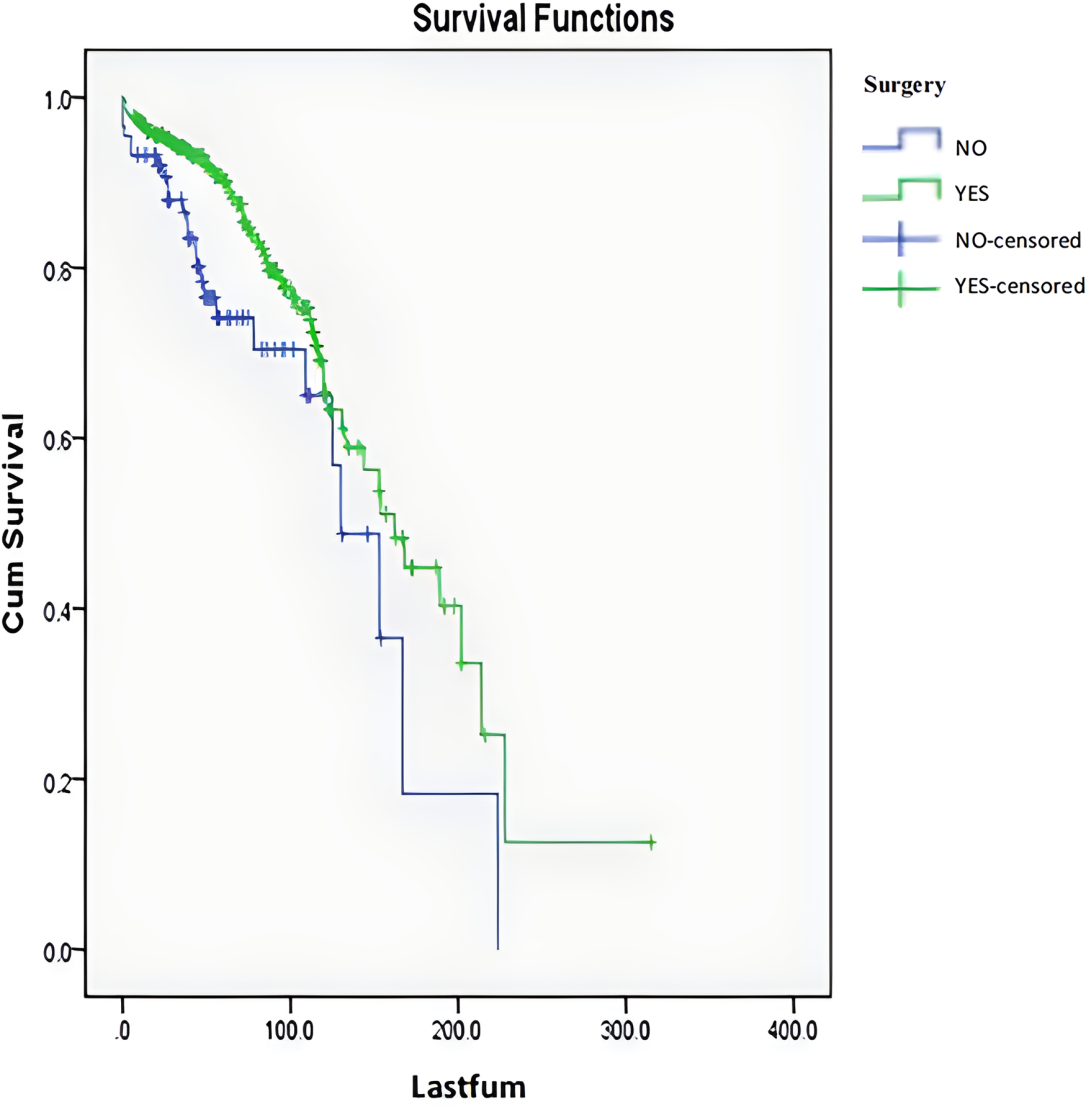
Survival curves of the patients with and without surgery.

A multivariate Cox time-dependent model analysis was used to analyze all variables, including significant variables in the univariate analysis, discretized age, ASIA score, and Enneking stage. The results revealed that surgical treatment [*P* = 0.002, HR = 0.431, 95% CI. (0.254, 0.729)], benign and malignant tumors [*P* < 0.001, HR = 2.788, 95% CI. (1.721, 4.516)], tumor and surrounding normal tissue boundary [*P* = 0.010, HR = 1.950, 95% CI. (1.171, 3.249)], and spinal instability [*P* = 0.031, HR = 1.731, 95% CI. (1.051, 2.851)] still had significant effects on survival. The survival curves for the surgical and non-surgical patients are shown in Figure 5.

The 5- and 10-year overall survival rates of the patients with different values for the four significant risk factors influencing survival are shown in Table 4.

**Table 4:** Associations of the 5- and 10-year overall survival rates of the patients with different values for the four significant risk factors influencing survival.

		5-year survival rate	10-year survival rate
Pathology	Benign	95.8%	91.3%
	Malignant	71.7%	36.9%
Tumor invasiveness	Confined	92.7%	71.1%
	Invasive	77.7%	56.2%
Motion-related pain	N	90.5%	66.9%
	Y	74.9%	58.1%
Surgery	N	74.1%	65%
	Y	90.1%	65.4%

### HRQoL and its influencing risk factors

Among the 187 surviving patients who were followed up by telephone, the mean follow-up time was 59 months. As shown in Table 5, the mean (standard deviation) scores of the five items in the EQ-5D questionnaire were 1.11 (0.34), 1.06 (0.29), 1.25 (0.46), 1.26 (0.46) and 1.18 (0.38), and the mean (standard deviation) total score was 5.84 (1.46).

**Table 5:** Summary of the HRQoL scores for the patients.

	HRQoL aspects	Mean	SD	% abnormal
EQ-5D-1	Ambulation	1.11	0.34	11%
EQ-5D-2	Self-care	1.06	0.29	6%
EQ-5D-3	ADL	1.25	0.46	24%
EQ-5D-4	Pain/Discomfort	1.26	0.46	26%
EQ-5D-5	Anxiety/Depression	1.18	0.38	19%
EQ-5D	Overall HRQoL	5.84	1.46	37%

By one-way ANOVA, it is showed that when the Enneking stage was classified into S1, S2–S3, and I–III, it was a significant risk factor for both overall HRQoL (EQ-5D total score) and various HRQoL components (walking ability, self-care ability, daily life, pain and discomfort, anxiety and depression) (*P* = 0.005, *P* = 0.001, *P* = 0.024, *P* < 0.001, *P* = 0.018, *P* = 0.048, respectively). When age was divided into three groups (<20, 20–50, >50 years), it was a significant risk factor for total EQ-5D score, daily life, pain and discomfort, and anxiety and depression (*P* < 0.001, *P* = 0.008, *P* < 0.001, *P* = 0.010, respectively). Previous surgical history for tumor lesions was a significant risk factor for walking ability (*P* = 0.021). Duration of disease was a significant risk factor for pain and discomfort (*P* = 0.033). Comparisons of the mean HRQoL scores corresponding to the significant risk factors at different values are shown in Table 6.

**Table 6:** Significant risk factors for quality of life corresponding to HRQoL scores at different values.

	EQ-5D		EQ-5D-1		EQ-5D-2		EQ-5D-3		EQ-5D-4		EQ-5D-5	
	Mean ± SD	*P* Value	Mean ± SD	*P* Value	Mean ± SD	*P* Value	Mean ± SD	*P* Value	Mean ± SD	*P* Value	Mean ± SD	*P* Value
Age												
<20	5.06 ± 0.31	*P *< 0.001					1.04 ± 0.19	*P *< 0.001	1.00 ± 0.00	*P *< 0.001	1.02 ± 0.14	*P *< 0.001
20–49	5.85 ± 1.27						1.24 ± 0.43		1.32 ± 0.49		1.19 ± 0.40	
50 and up	6.56 ± 1.71						1.47 ± 0.55		1.42 ± 0.50		1.31 ± 0.47	
Ennecking												
S1	5.26 ± 0.64	*P *< 0.001	1.00 ± 0.00	*P *< 0.001	1.00 ± 0.00	*P *= 0.002	1.08 ± 0.27	*P *< 0.001	1.13 ± 0.34	*P *= 0.003	1.05 ± 0.22	*P *= 0.013
S2&S3	5.65 ± 1.15		1.07 ± 0.26		1.03 ± 0.17		1.19 ± 0.39		1.22 ± 0.42		1.17 ± 0.38	
I&II	6.73 ± 1.80		1.28 ± 0.51		1.18 ± 0.45		1.53 ± 0.55		1.45 ± 0.55		1.30 ± 0.46	
Previous Surgery												
Y			1.44 ± 0.53	*P* = 0.001								
N			1.08 ± 0.30									
Duration												
Less than 1 year									1.18 ± 0.39	*P* = 0.007		
More than 1 year									1.39 ± 0.52			

## Discussion

Primary bone tumors often occur in young people aged 0–30 years, but only approximately 5% are primary tumors of the cervical spine. Some researchers believe that bone infarction, chronic osteomyelitis, Paget's disease, radiotherapy, and metal prosthesis use may be related to the occurrence of bone tumors (16), but there is currently no consensus. In recent years, molecular biology studies have shown that mutations of tumor suppressor gene p53, Receptor activator of nuclear factor-κΒ ligand (RANKL), osteoprotegerin, and other genes may be related to the pathogenesis of bone tumors (12). These findings provided a direction for research on related targeted drugs as alternative or adjuvant therapies (17, 18). Other researchers believe that the incidence of some bone tumors may be related to syndromic diseases (13). However, there were no diagnoses of syndromic diseases among the 341 patients in the present study.

The incidence rate of benign tumors was higher than that of malignant tumors. The real incidence rate of benign tumors would be even higher than the present findings, considering that most cases with asymptomatic benign tumors are not diagnosed, and many cases with clinically diagnosed benign tumors do not undergo surgery or biopsy (19). However, owing to the relatively complete construction of graded diagnoses and treatments in Germany, there is a referral bias in related research from large spinal tumor treatment centers, and the reported rates of malignant tumors are much higher, up to 85% (20). On the one hand, due to the differences in national conditions between China and Western Europe (20), the proportion of benign tumors in the present study was approximately 75%. Due to the imperfect construction of hierarchical diagnosis and treatment in China, the referral bias is relatively small. On the other hand, similar situations are found between China and the above countries. For example, Patients with primary spinal tumors are mostly first diagnosed in surgical treatment departments [mainly orthopedics in China, and both orthopedics and neurosurgery in Western Europe (20)/North America (21)], and further referred to auxiliary treatment departments if necessary. Therefore, the patient population in this study was characterized by small referral bias and also by referral mechanisms similar to the countries mentioned above, which make the patient population in this study epidemiologically representative.

The incidence rate of spinal tumors is low, and the pathological types are various. The clinical manifestations vary greatly and different structures can be affected. Although surgical resection is the main treatment method, there are few studies on the effects of surgery with satisfactory numbers of cases and follow-up periods, and thus it is difficult to comprehensively evaluate, understand, and compare the effects of different treatment methods. In addition, due to the long time span and continuous innovations in inspection methods, surgical procedures, and treatment concepts, it is difficult to make meaningful comparisons between recent cases and previous cases in a retrospective study. Therefore, it is extremely difficult to investigate curative effects for primary spinal tumors (22), and the treatment principles are mostly based on a summary of experience.

In a literature review, the present study found only five large case reports on primary spinal tumors (4, 7, 20, 23, 24). The sex ratios were 1:1, the mean ages were 55, 42, 43, 45, and 48 years, respectively, and the proportions of malignant tumors was 86%, 43%, 49%, 39%, and 100% respectively. The incidence of different types of tumors varied greatly among the previous reports, which may be related to differences in regions, races, and local medical referral systems. These factors often have significant impacts on research for rare diseases (4). Pain is the most obvious symptom of primary spinal tumors, with an incidence of >90%, and the pain is often increased during activities or at night (20). The pain may be localized to back in 60.2% or radicular in 24% cases (25).

The reported incidence of local palpable tumor mass is low, at 2.5% (20). while the incidence of SCI can reach 52% (20). A previous study found that the local recurrence rate for primary spinal tumors after resection was about 15%, and was closely related to the prognosis (all patients with malignant tumors died within 6 months of local recurrence), although correlation analyses showed that the tumor characteristics may have a greater impact on the early recurrence rate than the surgical procedures (20).

In previous large case studies on primary tumors of the spine, the proportion of the lesions in the cervical spine was relatively low, such as 14% (4), 17% (26), 24% (15), and 29% (20). And the lowest were at 7.3% (5). In our study involving 438 primary spinal tumor cases at the Department of Orthopedics, Peking University Third Hospital (27), primary cervical spinal tumor cases accounted for 57%, which was significantly higher than the proportion of foreign cases reported (4, 5, 15, 20, 26). Based on this, we carried out further research. This is the first large-scale case study of primary cervical spinal tumors. In the 341 cases, chordoma was the most common malignant tumor, consistent with previous findings (4, 27–29), while schwannoma was the most common benign tumor, consistent with some previous findings (23), but inconsistent with other findings (30). There was no significant sex difference between benign and malignant tumors, consistent with previous studies (15, 27). The mean age of the patients with benign tumors was 31.4 years, while the mean age of the patients with malignant tumors was 46.1 years, with a significant difference (*P* < 0.05). Meanwhile, the proportion of benign tumors was more than 10 times higher than the proportion of malignant tumors in patients aged <20 years, more than 5 times higher in patients aged 20–40 years, and similar in patients aged 40–50 years, while the proportion of malignant tumors was higher than the proportion of benign tumors in patients aged >50 years. These findings were similar to the conclusions in previous studies (4, 27). The primary diagnosis was fibrous dysplasia of bone in patients aged <10 years, osteoblastoma in patients aged 10–20 years, giant cell tumor of bone in patients aged 20–30 years, schwannoma in patients aged 30–60 years, and chordoma in patients aged >60 years, consistent with previous research conclusions(31). In terms of symptoms, previous studies (4, 27) reported that the incidence of spinal local pain was 79.4% and 77.6%, compared with 80.6% in the present study, and the incidence of neurological impairment was 31.1% and 45.2%, compared with 38.5% in the present study. Meanwhile, because of the relatively superficial characteristics of primary cervical spinal tumors, the incidence of spinal local masses in the present study was 11.2%, which was higher than that in previous reports (5, 27). The occurrence of nerve damage is related to the location of tumor involvement, as well as the age and instability of patients, but there is no consensus about the correlation (32). Previous studies have shown that upper cervical spinal tumors are mainly benign (26), which was further verified in the present study.

## Conclusions

In this cross-sectional study, we evaluated the survival period and medium and long-term health-related quality of life of patients with primary tumors of the cervical spine, and analyzed the significant related factors of tumor clinical characteristics. Surgery, myelopathy, malignancy, spinal pain relieved by lying down or supine position, and tumor infiltration on MRI were significant predictors for overall survival. Enneking stage and age were significant predictors for HRQoL.

## Data Availability

The raw data supporting the conclusions of this article will be made available by the authors, without undue reservation.
